# Supplementary material for: Accessibility of the unstructured α-tubulin C-terminal tail is controlled by microtubule lattice conformation
Source: bioRxiv. 2025 Sep 23:2025.09.23.678010. Preprint. [Version 1] doi: 10.1101/2025.09.23.678010 (PMC12485676; doi:10.1101/2025.09.23.678010)
Supplement: Supplement 1 [file NIHPP2025.09.23.678010v1-supplement-1.pdf]

bioRxiv preprint doi: https://doi.org/10.1101/2025.09.23.678010; this version posted September 23, 2025. The copyright holder for this preprint (which was not certified by peer review) is the author/funder, who has granted bioRxiv a license to display the preprint in perpetuity. It is made available under aCC-BY 4.0 International license.

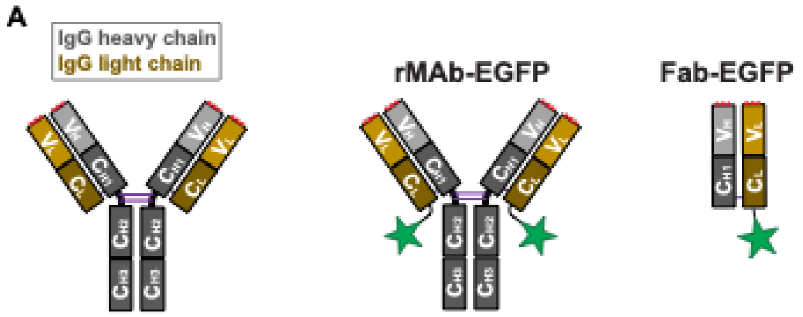

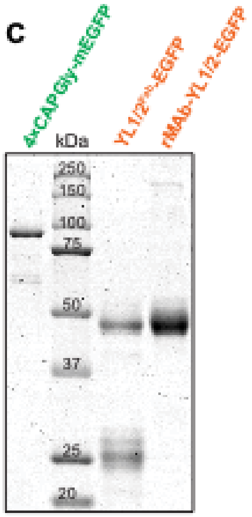

B

YL1/2 IgG (rat heavy chain gamma 2A, light chain kappa)

Heavy chain (440 aa):

```
EVQVLESGGGLVQPGNSLKLSCATSGFTFSSARMYWYRQFPEKRLEWVARIKAKSDNYATDYTESVKGRFTISRDDSKSS
IYLQMNNLKEEDTALYYCGWFDSGYDYWGQGVMVTVSSAETTAPSVYPLAPGTALKSNSMVTLGCLVKGYFPEPVTVTWN
SGALSSGVHTFPAVLQSGLYTLTSSVTVPSSTWSSQAVTCNVAHPASSTKVDKKIVPRECNPCGCTGSEVSSVFIFPPKT
KDVLTITLTPKVTCVVVDISQNDPEVRFSWFIDDVEVHTAQTHAPEKQSNSTLRSVSELPIVHRDWLNGKTFKCKVNSGA
FPAPIEKSISKPEGTPRGPQVYTMAPPKEEMTQSQVSITCMVKGFYPPDIYTEWKMNGQPQENYKNTPPTMDTDGSYFLY
SKLNVKKETWQQGNTFTCSVLHEGLHNHHTEKSLSHSPGK
```

Light chain (219 aa):

```
DIVLTQAPLSVSVTPGESASISCRSSKSLLRSKGITSLYWYLQRPGKSPQLLIYQMSNLASGVPDRFSSSGSETDFTLKI
SKVETEDVGVYYCGHRLEYPPTFGGGTKLELKRADAAPTVSIFPPSTEQLATGGASVVCLMNNFYPRDISVKWKIDGTER
RDGVLDSVTDQDSKDSTYSMSSTLSLTKADYESHNLYTCEVVHKTSSSPVVKSFNRNEC
```

Supp Figure 1. Recombinant YL1/2 antibody and purified probes.
(A) Schematic of a typical mammalian IgG molecule containing two heavy (H) and two light (L) chains. Light chains are comprised of one variable (V_L, light orange) and one constant (C_L, dark orange) region. Heavy chains are comprised of one variable (V_H, light gray) and three constant ($C_{H1–3}$, dark gray) regions. Red dots: complementarity determining regions (CDRs). Purple lines: disulfide bonds. rMAb-EGFP: recombinant monoclonal antibody (rMAb) with EGFP fused to the C-terminus of the light chain. Fab-EGFP: Fragment antibody binding (Fab) produced from rMAb-EGFP by papain cleavage.
(B) Experimentally-determined YL1/2 protein sequence. The deduced amino acid sequences of YL1/2 IgG heavy and light chains are shown. The red text indicates the CDRs involved in antigen recognition. Asterisks demarcate every 10 aa.
(C) Coomassie-stained SDS-PAGE gel of purified proteins.

bioRxiv preprint doi: https://doi.org/10.1101/2025.09.23.678010; this version posted September 23, 2025. The copyright holder for this preprint (which was not certified by peer review) is the author/funder, who has granted bioRxiv a license to display the preprint in perpetuity. It is made available under aCC-BY 4.0 International license.

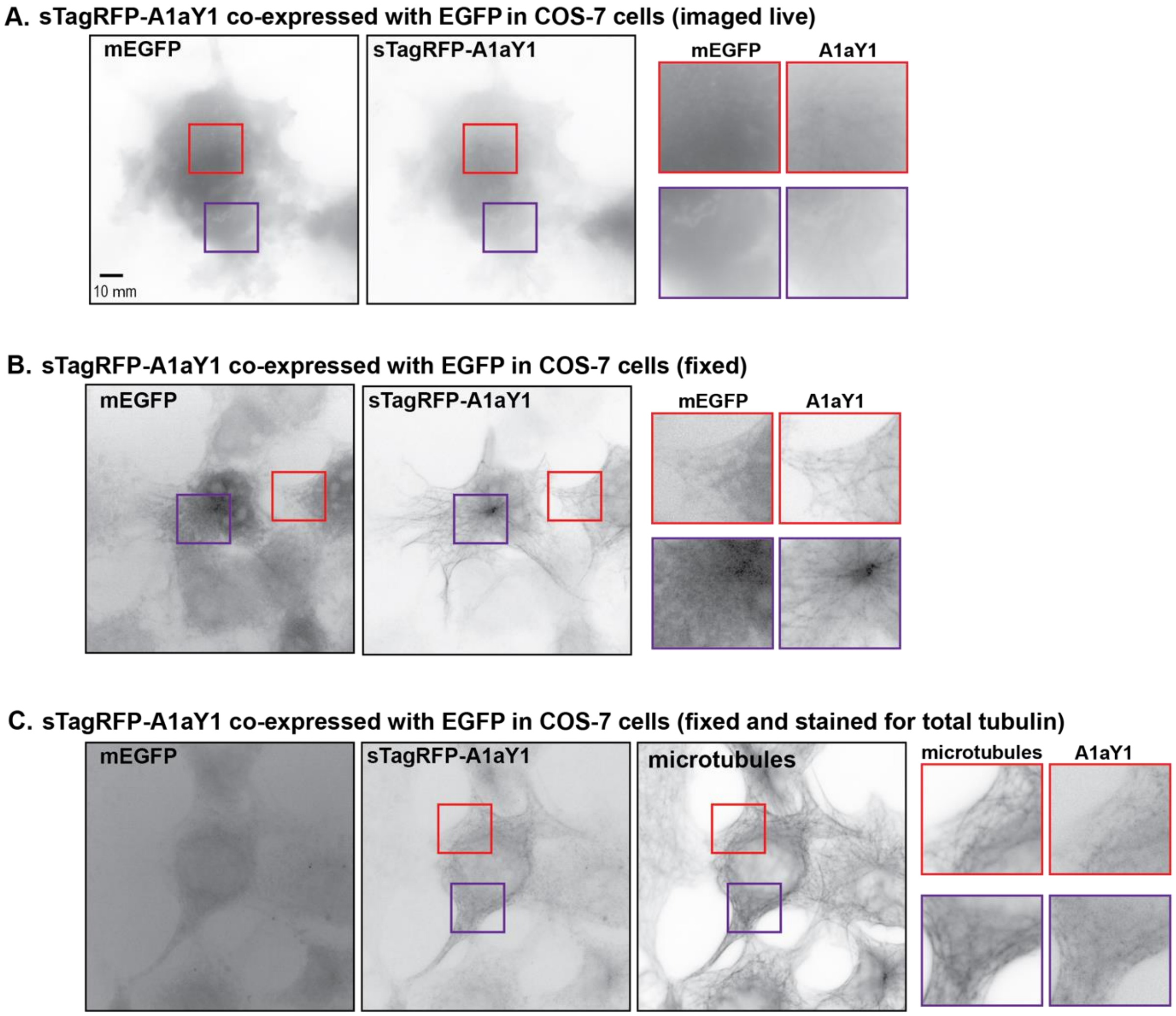

Supp Figure 2. A1aY1 sensor must be imaged in live cells.
COS-7 cells expressing sTagRFP-A1aY1 and mEGFP were (A) imaged live, (B) fixed and mounted, or (C) fixed and stained for total tubulin (microtubules). Scale bar: 10 μm. Magnified views of the red and purple boxed regions are shown to the right.

bioRxiv preprint doi: https://doi.org/10.1101/2025.09.23.678010; this version posted September 23, 2025. The copyright holder for this preprint (which was not certified by peer review) is the author/funder, who has granted bioRxiv a license to display the preprint in perpetuity. It is made available under aCC-BY 4.0 International license.

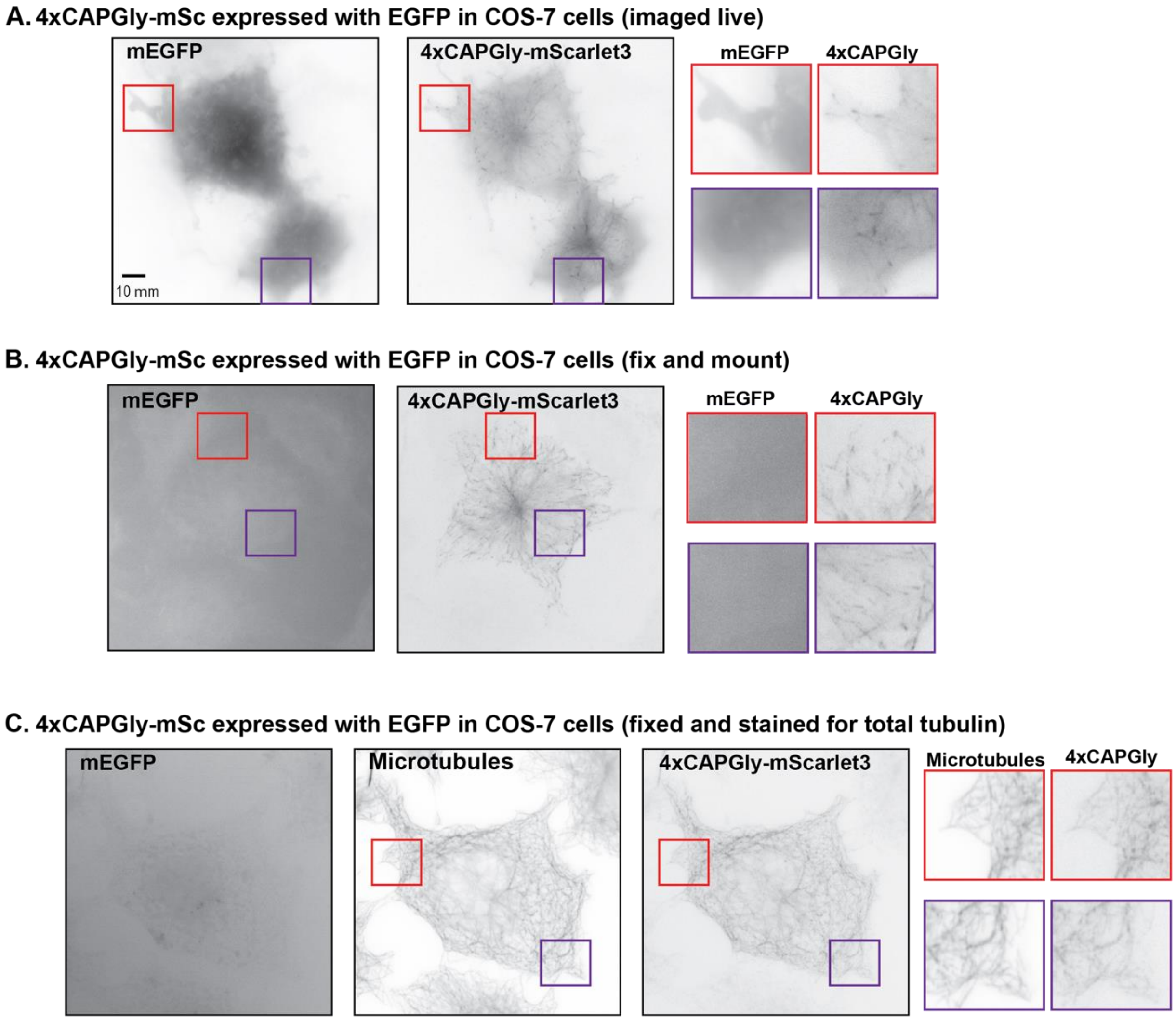

Supp Figure 3. 4xCAPGly sensor must be imaged in live cells.
COS-7 cells expressing 4xCAPGly-mSc3 and mEGFP were (A) imaged live, (B) fixed and mounted, or (C) fixed and stained for total tubulin (microtubules). Scale bar: 10 μm. Magnified views of the red and purple boxed regions are shown to the right.

bioRxiv preprint doi: https://doi.org/10.1101/2025.09.23.678010; this version posted September 23, 2025. The copyright holder for this preprint (which was not certified by peer review) is the author/funder, who has granted bioRxiv a license to display the preprint in perpetuity. It is made available under aCC-BY 4.0 International license.

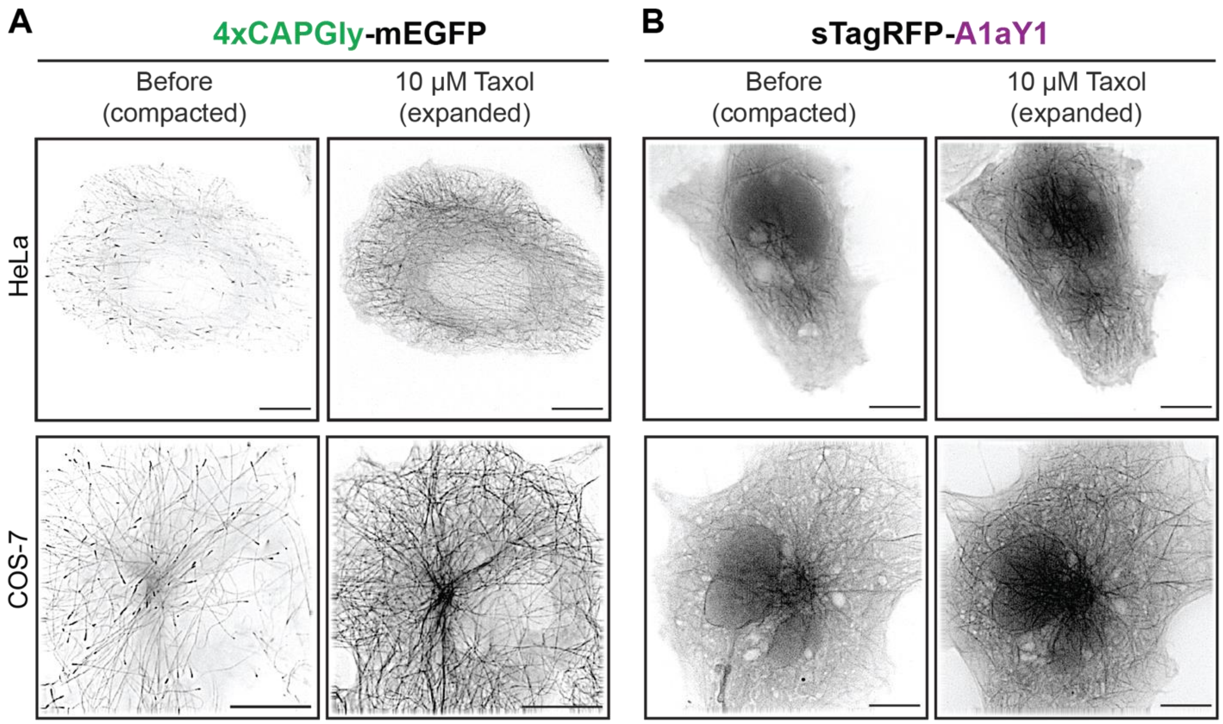

Supp Figure 4. Transiently-expressed probes bind to the microtubule lattice after Taxol expansion.
Representative images of (A) 4xCAPGly-mEGFP or (B) sTagRFP-A1aY1 probes imaged live in (top) HeLa or (bottom) COS-7 cells before or after addition of 10 μM Taxol. Scale bars: 10 μm.

bioRxiv preprint doi: https://doi.org/10.1101/2025.09.23.678010; this version posted September 23, 2025. The copyright holder for this preprint (which was not certified by peer review) is the author/funder, who has granted bioRxiv a license to display the preprint in perpetuity. It is made available under aCC-BY 4.0 International license.

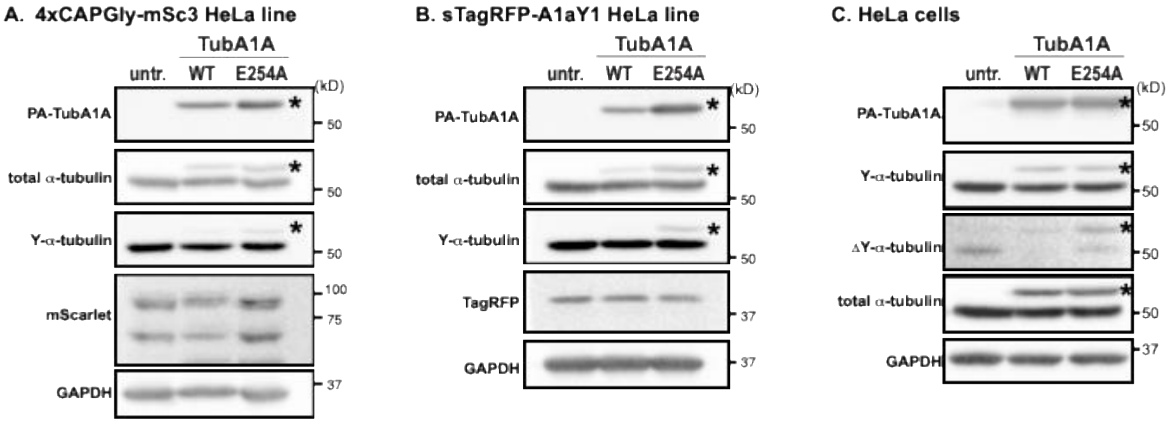

Supp Figure 5. Western blot of HeLa cells expressing internal PA-tagged tubulin. Whole cell lysates were prepared from (A) 4xCAPGly-mSc3 stable HeLa cells, (B) sTagRFP-A1aY1 stable HeLa cells, or (C) HeLa cells. In all cases, the cells were untransfected (untr.) or transfected with plasmids for expressing PA-tagged WT or E254A α-tubulin (TubA1A). Asterisks denote upshifted PA-tagged tubulin bands.

bioRxiv preprint doi: https://doi.org/10.1101/2025.09.23.678010; this version posted September 23, 2025. The copyright holder for this preprint (which was not certified by peer review) is the author/funder, who has granted bioRxiv a license to display the preprint in perpetuity. It is made available under aCC-BY 4.0 International license.

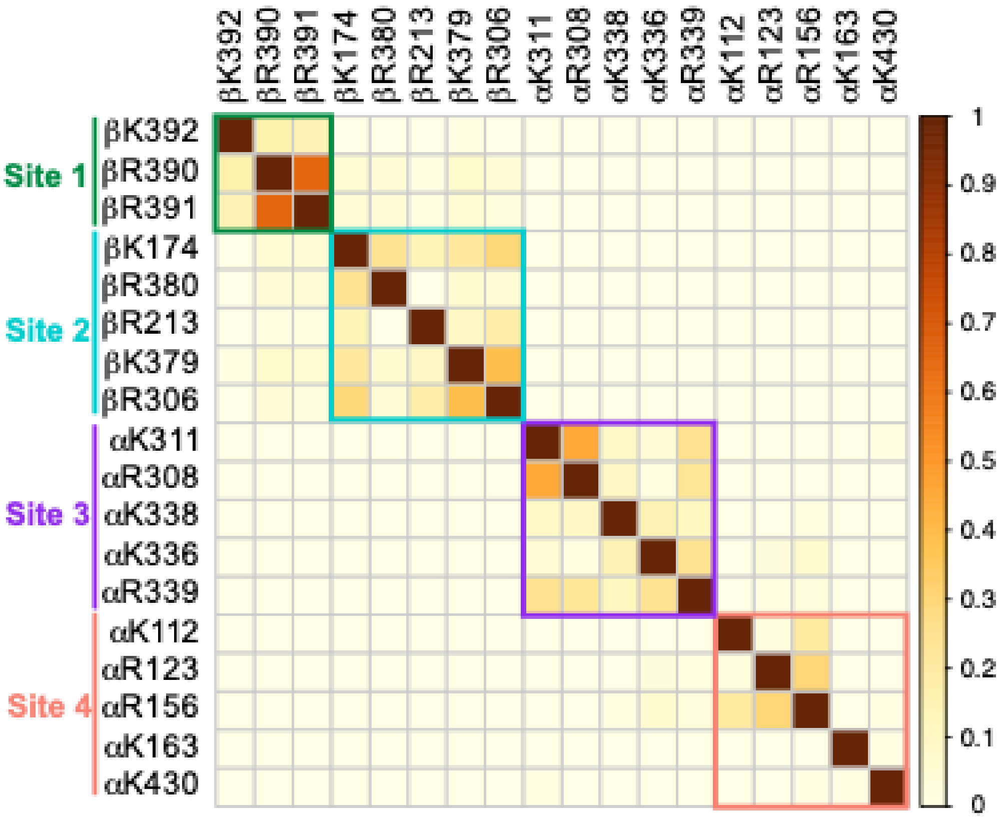

Supp Figure 6: The Y-αCTT primarily contacts four sites along a GDP microtubule lattice. Jaccard index plot indicating the frequency of two residues simultaneously forming salt bridges with the Y-αCTT based on MD simulations of a GDP microtubule lattice**.** The scale represents the Jaccard index where an index of 0 indicates that the two residues are never interacting with the Y-αCTT at the same time and an index of 1 indicates that when one of the two residues is interacting with the Y-αCTT, the other is also interacting with the Y-αCTT.

bioRxiv preprint doi: https://doi.org/10.1101/2025.09.23.678010; this version posted September 23, 2025. The copyright holder for this preprint (which was not certified by peer review) is the author/funder, who has granted bioRxiv a license to display the preprint in perpetuity. It is made available under aCC-BY 4.0 International license.

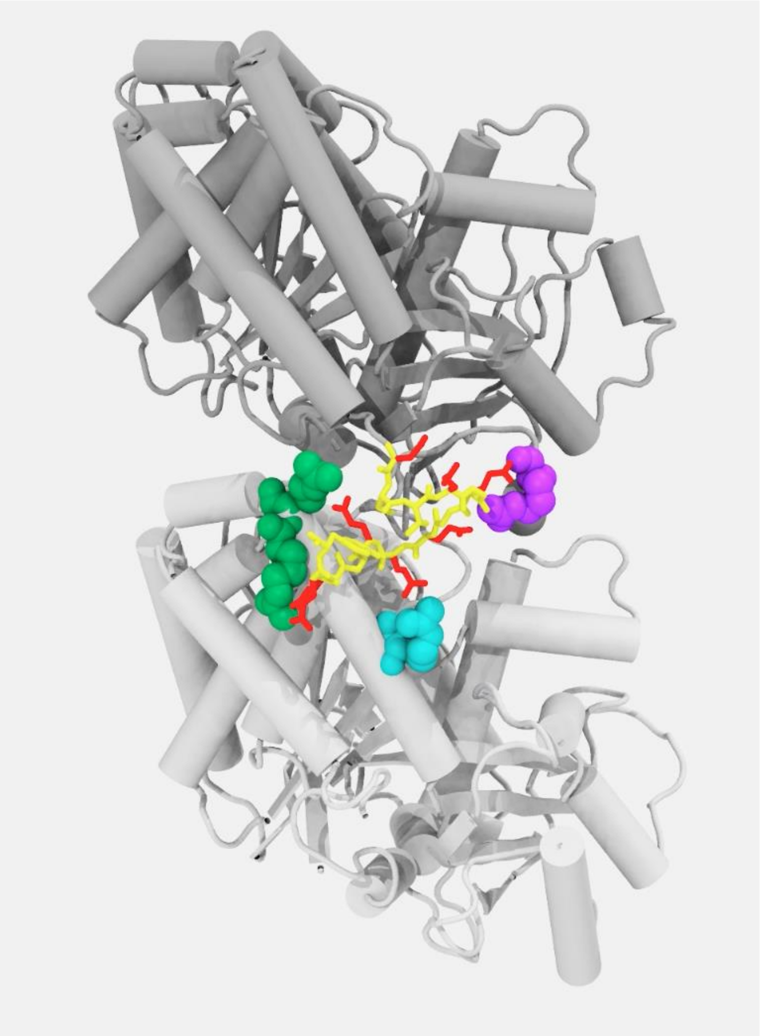

Supp Movie 1. Movie from MD simulations.
Representative movie from MD simulations showing interactions of the αCTT with the microtubule body over 240 ns. The tubulin body is shown in cartoon and colored gray. The αCTT is shown in stick and colored yellow with the glutamate sidechains in red. The residues in site 1 (green), site 2 (cyan), and site 3 (magenta) appear as spheres when the αCTT is forming salt bridges with those residues.

bioRxiv preprint doi: https://doi.org/10.1101/2025.09.23.678010; this version posted September 23, 2025. The copyright holder for this preprint (which was not certified by peer review) is the author/funder, who has granted bioRxiv a license to display the preprint in perpetuity. It is made available under aCC-BY 4.0 International license.

Supplementary Table 1. Primers used in cloning

CAPGLY_for1	CTGCAGTCGACGGTACCGCGGGCCCGGATGCTGAAACCCAGCGGGCTG
CAPGLY_rev1	CACCTCCCGATCCACCACCGCCCCTCTGGAGTTTGTCAGCTTTGGTCTTTTC
CAPGLY_for2	GTACCGCGGGCCCGGGATCCAATG CTGAAACCCAGCGGGCTG
CAPGLY_revGFP	GCCTGCACCTGAGTGTTTACTTTTACTTGTACAGCTC GTCCATGC
CAPGLY_revSc3	CTGCACCTGAGTGTTTACTTTTAGGAGCCACCGGAGCC
CAPGLY_for3	GCGGCAGCCA TATGCTCGAGCTGAAACCCAGCGGGCTGAAG
CAPGLY_rev3	CTTTCGGGCTTTGTTAGCAGCCG TTACTTGTACAGCTCGTCCATGC
A1aY1_for1	GATCTCGAGCTCAAGCTTCGGCAACAGTCAAGTTCAAATAC
A1aY1_rev1	CGCGGTACCGTCGACTGCAGTTACTTCTTCTGCTTCTCC
A1aY1_for2	TACCGCGGGCCCGGGATCCAATGGTGAGCAAGGGCGAG
A1aY1_rev2	CTGCACCTGAGTGTTTACTTTTACTTCTTCTGCTTCTCCAGC
A1aY1_for3	GCGGCAGCCATATGCTCGAGATGGCAACAGTCAAGTTCAAATAC
A1aY1_rev3	CCATGGTACCCTTCTTCTGCTTCTCCAG
sTagRFP_for1	GCAGAAGAAGGGTACCATGGTGAGCAAGGGCGAG
sTagRFP_rev1	TCGGGCTTTGTTAGCAGCCGTTACTTGTACAGCTCGTCCATG
His-PA_for	TGCCGAAGATGATGTGGTGGGGGGAGGAGATGATTCCTTC
His-PA_rev	CCTGGCATGGCAACGCCAATGGTCTTGTCACTTGGCATC
IRES-GFP_removal_for	AGCGGCCGCAATTCACTCCTC
IRES-mEGFP_removal_rev	TTAGTATTCCTCTCCTTCTTC
mEGFP-TubA1A_for	CTCAAGCTTCGAATTCTGCAATGGTGAGCAAGGGCGAG
mEGFP-TubA1A_rev	AGGAGTGAATTGCGGCCGCTTTAGTATTCCTCTCCTTCTTCCTCAC
pCIG2_rev	TGCAGAATTCGAAGCTTGAGC
PA-mEGFP-N1_for1	GGTGGAGGCGGTTCAGGC
PA-mEGFP-N1_rev1	CACCACATCATCTTCGGCACC
MAP2_for	GTGCCGAAGATGATGTGGTGATGGCAGATGAACGGAAAG
MAP2_rev	CCGCCTGAACCGCCTCCACCCAAGCCCTGCTTAGCGAG
MmMAP7_for	GTGCCGAAGATGATGTGGTGGCGGAGCAGGGAGCTGGC
MmMAP7_rev	CCGCCTGAACCGCCTCCACCTATAACTTCTGCGGTCTGTTGTGTCTGCAC
PA-mEGFP-N1_for2	GGCGTTGCCATGCCAGGTGCCGAAGATGATGTGGTGAGCAAGGGCGAGGAGCTGTTC
PA-mEGFP-N1_rev2	CATGGTGGCGACCGGTGG
Kif5C_for	ATCCACCGGTCGCCACCATGGCGGATCCAGCCGAATGC
Kif5C_rev	GCACCTGGCATGGCAACGCCCTCGAGCGGATCCCGGGC
tau_for	GATCTCGAGCTCAAGCTTCGATGGCTGAGCCCCGCCAG
tau_rev	CGCGGTACCGTCGACTGCAGTCACAAACCCTGCTTGGCCAG
CAMSAP2_for	GATCTCGAGCTCAAGCTTCGATGGGGGATGCTGCAGACCCCA
CAMSAP2_rev	CGCGGTACCGTCGACTGCAGCTATGCCTTAGTGGGTAAAAGTTTTTTGG
CAMSAP3_for	GATCTCGAGCTCAAGCTTCGATGGAGATCAAGTCGCTGGACCA GTACGATTTCTCGCGG
CAMSAP3_rev	CGCGGTACCGTCGACTGCAGCTATTTGGGAGTGCCGCC
pCAGGS_for	AGCGGCCGCAATTCACTCCTC
pCAGGS_rev	AATTCGAAGCTTGAGCTCGAG
PA-mEGFP_MAP_for	TCGAGCTCAAGCTTCGAATTATGGGCGTTGCCATGCCA
PA-mEGFP_MAP_rev	AGGAGTGAATTGCGGCCGCTTTATCTAGATCCGGTGGATCCCG
PA-MAP2-mEGFP_rev	AGGAGTGAATTGCGGCCGCTTTACTTGTACAGCTCGTCCATGC
